# Comparing Treatment Outcomes Between In-Hospital and Emergency Department Management of Patients With Transient Ischemic Attacks

**DOI:** 10.7759/cureus.20261

**Published:** 2021-12-08

**Authors:** Roger S Taylor, Nnennaya U Opara, Joshua Burg

**Affiliations:** 1 Emergency Medicine, Charleston Area Medical Center, Charleston, USA; 2 Emergency Medicine, CAMC Education and Research Institute, Charleston Area Medical Center, Charleston, USA

**Keywords:** abcd2 score, ecg, mri, ct, ed, thrombolytics, anti-lipids, transient ischemic attack

## Abstract

Introduction

A transient ischemic attack (TIA) is a medical emergency, as it is a sudden neurological episode caused by ischemia in a vascular territory in the brain, which lasts less than one hour. TIA definition has shifted from time-based to tissue-based according to modern literature. It is considered a warning sign for an impending stroke. Symptoms could range from weakness on one side of the body, diaphoresis, to slurred speech. In this study, we examined the differences in health outcomes, when patients diagnosed with TIA are treated and discharged home from the ED, versus when admitted to the hospital for additional care.

Methods

This is a descriptive and retrospective study. We examined all patients’ encounters from January 1, 2018 to December 31, 2019 at four emergency department locations. The cohort compared patients diagnosed with a TIA who takes medications (anti-lipid, antiplatelet drugs) versus patients diagnosed with a TIA who are not on any preventive medication. We compared the hospital readmission rate between these two group of patients and the need for additional medical treatments. Our study also considered hospital length of stay (LOS), admission rate, and its impact on patients with comorbidities.

Results

There were 983 patients included in the study. The patients on TIA prophylactic medications prior to coming to the ED made up (60.7%), and (51.2%) in this group required additional medications during hospital admission. The remaining 162 (39.3%), p=0.001 patients, were not on TIA prophylactic medications prior to presenting in the ED. The patients who required additional medications while in the ED were significantly older (mean +/-SD, 68.6 +/-14.0 years versus 62.18 +/- 17.4 years, p=0.001). Following a multivariate analysis, age greater than 60 (CI: 3.52-3.91, p=0.001) and results of the head CT/MRI investigations for any signs of neurological damage, were all found to be independent predictors of longer hospital stay and treatment outcomes. There were no significant differences in the treatment outcome for patients with TIA based on longer hospital stay and extra medication administration in the ED.

Conclusion

In our study, we observed that approximately, 75% of the patients who were on TIA prophylactic medications prior to presenting in the ED with symptoms of TIA were admitted to the hospital for further monitoring, compared to other group of patients who were not on TIA medications. We did also noted that there were no differences in mortality outcome between patients treated and discharged from the ED, versus patients admitted to the hospital for additional treatment. Lastly, patients who are 68 years and older, made up two-thirds of patient population admitted in the hospital and required additional medications, compared to younger patients.

## Introduction

Transient ischemic attacks (TIA) and strokes are disease processes with major implications for societies and the healthcare organizations. Annually, 795,000 individuals are diagnosed with a TIA/stroke, which is the equivalent of one being diagnosed every 40 seconds in the United States [1]. In the state of West Virginia, heart disease is the leading cause of death, and the state is ranked ninth highest death rate from cardiovascular disease in the country [2]. Majorities of TIA are admitted through the four emergency department (ED) hospitals in our region that puts a huge burden on inpatient services and on the hospital emergency department. Studies that include clinical decision tools have been developed to weigh the need to either admit TIA patients to the hospital for further workup or to discharge home from the emergency department [3]. The definition of TIA has changed with advances in learning gained from evidence-based medicine. Historically, a TIA was defined as brief episode of neurological dysfunction resulting from focal cerebral ischemic event with symptoms lasting < 24 hours [4]. A more apt definition of TIA at this point becomes a transient episode of neurological dysfunction involving the brain, spinal cord, or retinal ischemia without acute infarction [5]. Through an in-depth understanding of TIA, clinicians have discovered that a TIA could be more insidious than previously believed, and should be likened to “unstable angina” as predictors of likely future vascular event [6].

Any patients presenting to the ED with a diagnosis of TIA have symptoms either actively resolving, or are resolved on presentation. Standard of care begins with a quick history and physical exam, including a full neurology exam and NIH stroke scale evaluation along with an initial glucose test [7]

The ABCD2 score (A=age > 60 years/ score 1; B= SBP>140 mmHg or DBP >90 mmHg/ score 1; C= clinical features: speech impairment without weakness/ score 1. Weakness with/without speech impairment/score 2; D= duration: > 60 minutes/score 2, between 10 and 59 minutes/score 1; D= diabetes/score 1) is a very essential tool we use in the ED to predict future events related to strokes/TIA. Patients with ABCD2 score between 6 and 7, have an 8% stroke risk within 48 hours, while patients with an ABCD2 score below 4, have a 1% risk of stroke within 48 hours [8]

In this study, we compared treatment outcomes for TIA patients treated and discharged from the ED, versus TIA patients who were admitted in the hospital for further treatment, to evaluate their subsequent work ups in a retrospective fashion, and evaluate the possibility of an outpatient referral system for these workups in these patient population to see if such management, is appropriate for patient safety and cost effectiveness standpoint. Our next goal was to see if the improvement of patients’ outcomes, was based on medication changes or dose adjustments, and whether admitting them to the hospital, appeared to be truly necessary.

## Materials and methods

The study was a descriptive, retrospective cohort study. Data were extracted for review inclusive of the period of January 1, 2018 and December 31, 2019. We reviewed a total of 985 patients who met the inclusion criteria. The remaining 162 (39.3%) patients are those who were not on TIA prophylactic medications prior to admission in the hospital. Charleston area Medical Center Institutional Review Board, issued approval 21-777 for the study.

Inclusion criteria were patients, ages >18 years, patients on anti-lipid and antihypertensive drugs, and patients diagnosed or suspected of TIA either in the emergency department or inpatient setting by the ED physician. Exclusion criteria were patients with ischemic stroke on CT/CTA/MRI imaging in the ED, patients less than age 18, trauma patients and pregnant patients. Results of MRI, echo, Carotid duplex and inpatient documentation of all services were reviewed. This information helped in determining whether or not, it was necessary admitting every patient with TIA, and if that improved overall health outcome. Additionally, we identified any further neurological events that occurred during admission such as CVA, or new episodes of TIA. Categorical variables such as gender and age, were analyzed as descriptive statistics and reported as means, and in percentage. Continuous variables are analyzed using a two-sample t-test. The cohorts was compared by chi-square to determine if there were any difference in outcomes. These multivariate analysis were used in determining the impact of all variables on patients overall health outcomes. The Statistical Package for Social Sciences (SPSS) technique was utilized to gain deeper understanding of the data in relation to clinical features of stroke. The variables were selected for the purpose of finding its correlation to developing TIA/stroke with p-value < 0.05 considered significant.

Data collection

Relevant data were extracted from patients’ electronic health records. Patients’ age, gender, race, BMI, vital signs, medication history, medical interventions and pre-existing health conditions such as diabetes and neurological events, date of admission to hospital, and hospital length of stay were reviewed using a designated International Classification of Disease (ICD) code.

Statistical analysis

Data analysis was performed using SPSS, and is expressed as number (percentage) or median (IQR), except otherwise indicated. Descriptive statistics, such as means and standard deviations for continuous variables and proportions and frequencies for categorical variables, were used to analyze patient characteristics. Frequency comparisons were performed using two-sample t-test for continuous values and Chi-square test for categorical variables. A p-value of <0.05 was used in determining statistical significance.

## Results

From January 2018 through December 2019, a total of 983 patients at three hospital sites were included in the cohort, of whom 821 (60.7%) patients were on TIA prophylactic medications prior to coming to the ED, while 162 (39.3%) patients are those who are not on TIA prophylactic medications prior to admission in the hospital. All patients who came to the emergency department with neurological symptoms were systematically examined for TIA upon discharge from hospital with head CT scan to rule out any suspicion of ischemic, or hemorrhagic stroke. We compared ED 90-day health outcomes to inpatient 90-day outcomes for these patients, particularly patients with severe comorbidities such as diabetes and hypertension (Table 1).

**Table 1:** Comparing patients’ 90-day outcomes both in ED and inpatient care. ED: emergency department; TIA: transient ischemic attack.

Patient demographic	ED 90-day outcomes OR 95% CI	p-value	ED Recurrent TIA in 90 days OR (95% CI)	p-value	Inpatient 90-day outcomes OR (95% CI)	p-value	Inpatient 90-day Recurrent TIA (95% CI)	p-value
Age	1.25(3.91-8.83)	0.001	2.01(1.99-2.02)	0.62	2.12(2.1-2.14)	0.001	2.01(1.99-2.02)	0.84
Diabetes	1.56(1.2-2.04)	0.14	2.38(1.92-2.98)	0.24	1.66(1.4-2.01)	0.10	2.21(1.89-2.65)	0.45
Hypertension	3.21(1.56-4.86)	2.27	2.76(2.1-3.66)	1.68	2.61(2.01-3.38)	0.93	3.05(2.42-3.83)	2.60

The patients who required additional medications while in the ED were significantly older (mean +/-SD, 68.6 +/-14.0years.p=0.001) as illustrated in (Table 2). 

**Table 2:** Age group statistics. TIA: transient ischemic attack.

TIA prophylaxis	Total	Mean age	Standard deviation	Standard error mean	95% CI	p-value
Yes	821	68.55	13.991	0.488	1.254(3.91-8.83)	0.001
No	162	62.18	17.327	1.361	1.446(3.521-9.22)	0.001

**Figure 1:**
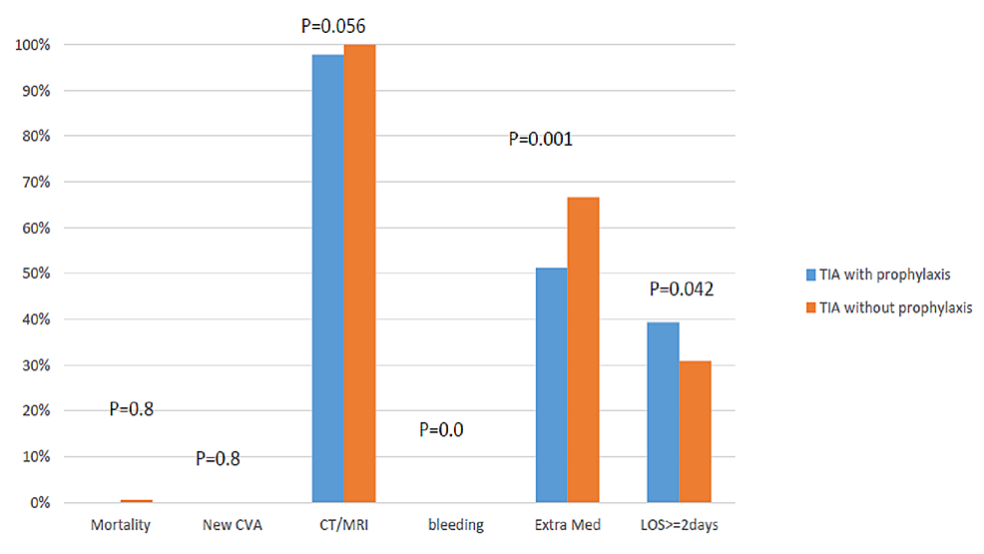
Patients' outcome comparison in ED and inpatient care. CVA: cerebrovascular accident; CT: computed tomography; ED: emergency department; LOS: length of stay; TIA: transient ischemic attack.

Following a multivariate analysis (Table 3), age greater than 60 (CI: 3.52-3.91, p=0.001), and results of the head CT/MRI investigations for any signs of neurological outcome were found to be independent predictors of longer hospital stay and treatment outcomes as shown in Figure-1]. Head CT (97.2%), and thrombolytic therapy (89%) were the most reported ED interventions. Furthermore, over (87%) of the patients with TIA were admitted. There was no significant difference in the treatment outcome for patients with TIA based on longer hospital stay and extra medication administration in the ED. Additionally, patients who were on TIA prophylaxis prior to their ED visit, spent two or more days in the hospital (60.7%) compared to patients who were not on TIA prophylaxis (39.3%), with a median length of hospital stay of two days (p=0.02) (Figure 1).

The two most frequent comorbidities requiring additional medication while in the hospital were obesity (69.8%) and hypertension (57%) (Table 1). Female patients made up two-thirds of all patients who presented in the ED with symptoms of TIA during our study period. The two most common complaints our patients had were extremity weakness (57%) and slurred speech (47%).

We subsequently, focused our investigations on the patients who had the most comorbidity, and are over the age of 68 as they made up 59% of our sample size (Table 2). There was no difference in mortality rate between these groups of patients. During our study period, a total of five patients died from stroke complications. All the patients who presented in the ED with TIA received imaging studies (CT scans, MRI, MRA), and no abnormalities were seen in their scan results. Sensitivity analyses focused only on patients who underwent brain imaging, and were observed for 90 days.

## Discussion

These results support the association of guidelines and protocols necessary for improved outcomes for patients with TIA [9]. The main protocols for TIA management (i.e., CT scan, carotid artery imaging, antihypertensive medication, Statin therapy, thrombolytic therapy, and antiplatelet), can all be administered routinely at various medical centers. However, patients with more complex neurological symptoms should be referred to neurologists for more specialized interventions [10]. Our study shows that there are no differences in patients’ outcome regarding types of treatments for TIA or ischemic stroke, and that each case needed to be approached with the guideline-concordant processes of care for which they are eligible (Figure 1). Patients who presented to our ED with symptoms of TIA were screened for other comorbidities that contribute to the development of stroke, such as obesity, hypertensive heart disease, and other prescription medications they might be on like blood thinners (Table 3). Meta-analysis data has shown that patients who showed improved health outcomes were those who received treatment for carotid stenosis, hyperlipidemia, thrombolytic and antihypertensive medication, prior to developing TIA, compared to patients who had no TIA prophylactic treatment. These could be explained due to the fact that patients with comorbidities such as hypertension, diabetes, etc., are generally prescribed TIA prophylactic medications, which prevent severe neurological consequences of stroke, compare to patients who are not on these prophylactic medications. Our study shows that these group of patients (patients on TIA prophylactic medications) make up two-thirds of our study population (Table 3). These data further support the AHA/ASA secondary prevention recommendations [11].

**Table 3:** Patient demographics. TIA: transient ischemic attack; SD: standard deviation.

Variable	Total	SD (%)	TIA with prophylaxis	SD (%)	TIA without prophylaxis	SD (%)	p-values
Obesity	662	67.3	573	69.8	89	54.9	0.001
Diabetes	297	30.2	280	34.1	17	10.5	0.001
Hypertension	545	55.4	468	57.0	77	47.5	0.001
Extra meds	528	53.7	420	51.2	108	66.7	0.001
Male	434	44.2	360	43.8	74	45.7	0.667
Female	549	55.8	461	56.2	88	54.3	0.667

The TIA registry.org project (2009-2011), reported a 90-day recurrent stroke rate of 3.7%, a one-year recurrent stroke rate of 5.1% and a one-year mortality rate of 1.8%, among patients who received early stroke specialist care [12]. We observed that almost all patients who presented to our ED with TIA, were admitted to the hospital. Next, we conducted sensitivity analyses on patients who were older (68 years and older), for whom we had an extensive medical history of their health care utilization; the results of the analysis supported the overall findings [Table-2].

Although several risks prediction scores have been validated for patients with strokes and TIA, we did not base our study criteria solely on the scores. Reason was as a result of insufficient data available for retrieval in electronic health records to calculate the ABCD2 score. The scores were derived directly from patients’ notes, and so we based our analysis on the information contained in the EHR since this was a retrospective study. However, we did compare overall patients’ 90-day outcomes when treated in ED and discharged home versus when admitted. Our study showed no significant difference in patients’ outcomes (Table 1).

A recent systematic study and meta-analysis (33 studies, 16,070 patients) of the ABCD2 scores and stroke risks did not classify ABCD2 </>4. They concluded that the score simply lead to small revisions of baseline stroke risk specifically in settings of very low risk and when used by other clinicians but Neurologists, although most of their data were derived from specialists [13].

The major challenges that hinder the smooth transition of pre-clinical research into successful therapeutic drug selection include confounding diseases like hypertension, diabetes, and hypercoagulable genetic disorders, age and gender effects in stroke patients, development of medical devices, investigating medical conditions that accompanied stroke episodes, reproducibility of pre-clinical stroke research data and modelling functional and behavioral outcome [14].

Since this was a retrospective study, we cannot ignore the possibility of confounding. We observed that patients’ age played a significant role in health outcomes. Hence, the older the patient, the sicker they become.

Limitations

In addition to other potential confounders in the study (comorbidity and age), the study population only reflects the demographic characteristics of the local population, and so cannot be generalized. The study could also be expanded using a larger sample size with inclusion of more variables such as one-year post TIA outcomes and other health comorbidities as this study may not have included the strongest associations with outcomes. Future studies might consider identifying most of the ABCD2 score components experientially.

## Conclusions

Patients who present to the ED with symptoms of TIA need not to be admitted to the hospital, except when such patient symptoms fail to resolve in the emergency room. All patients treated for TIA need continuous outpatient care with a neurologist, to achieve a full recovery. We also observed that although the use of CT, ECG and thrombolytic agents in the ED was high, however, neurology consults in the ED for all patients with symptoms of cerebrovascular events do have a significant impact on overall patient outcomes.

## References

[1] Albers GW, Caplan LR, Easton JD, Fayad PB, Mohr JP, Saver JL, Sherman DG (2002). Transient ischemic attack--proposal for a new definition. N Engl J Med.

[2] Association Association, A. H. (2017 (2021). Division of Health promotion and Chronic Disease. https://dhhr.wv.gov/hpcd/data_reports/Pages/Fast-Facts.aspx.

[3] Lesenskyj AM, Maxwell CR, Veznedaroglu E, Liebman K, Hakma Z, Binning MJ (2016). An analysis of transient ischemic attack practices: Does hospital admission improve patient outcomes?. J Stroke Cerebrovasc Dis.

[4] Easton JD, Saver JL, Albers GW (2009). Definition and evaluation of transient ischemic attack: a scientific statement for healthcare professionals from the American Heart Association/American Stroke Association Stroke Council; Council on Cardiovascular Surgery and Anesthesia; Council on Cardiovascular Radiology and Intervention; Council on Cardiovascular Nursing; and the Interdisciplinary Council on Peripheral Vascular Disease. The American Academy of Neurology affirms the value of this statement as an educational tool for neurologists. Stroke.

[5] Sanders LM, Srikanth VK, Blacker DJ, Jolley DJ, Cooper KA, Phan TG (2012). Performance of the ABCD2 score for stroke risk post TIA: meta-analysis and probability modeling. Neurology.

[6] Bravata DM, Myers LJ, Reeves M (2019). Processes of care associated with risk of mortality and recurrent stroke among patients with transient ischemic attack and nonsevere ischemic stroke. JAMA Netw Open.

[7] (2021). Stroke Statistics. Retrieved from National Center for Chronic Disease Prevention and Health promotion, Division for Heart Disease and Stroke Prevention. https://www.cdc.gov/stroke/facts.htm.

[8] Deplanque D, Bastide M, Bordet R (2018). Transient ischemic attack and minor stroke: definitively not so harmless for the brain and cognitive functions. Stroke.

[9] Siket MS, Cadena R (2021). Novel treatments for transient ischemic attack and acute ischemic stroke. Emerg Med Clin North Am.

[10] Li X, Zhou G, Zhou X, Zhou S (2013). The efficacy and safety of aspirin plus dipyridamole versus aspirin in secondary prevention following TIA or stroke: a meta-analysis of randomized controlled trials. J Neurol Sci.

[11] Kleindorfer DO, Towfighi A, Chaturvedi S (2021). 2021 guideline for the prevention of stroke in patients with stroke and Transient ischemic Attack: A guideline from the American heart Association/American Stroke Association. Stroke.

[12] Amarenco P, Lavallée PC, Labreuche J (2016). One-year risk of stroke after transient ischemic attack or minor stroke. N Engl J Med.

[13] Wardlaw JM, Brazzelli M, Chappell FM, Miranda H, Shuler K, Sandercock PA, Dennis MS (2015). ABCD2 score and secondary stroke prevention: meta-analysis and effect per 1,000 patients triaged. Neurology.

[14] Kuriakose D, Xiao Z (2020). Pathophysiology and treatment of stroke: present status and future perspectives. Int J Mol Sci.

